# Identification and monitoring of mutations in circulating cell-free tumor DNA in hepatocellular carcinoma treated with lenvatinib

**DOI:** 10.1186/s13046-021-02016-3

**Published:** 2021-06-26

**Authors:** Yasutoshi Fujii, Atsushi Ono, C. Nelson Hayes, Hiroshi Aikata, Masami Yamauchi, Shinsuke Uchikawa, Kenichiro Kodama, Yuji Teraoka, Hatsue Fujino, Takashi Nakahara, Eisuke Murakami, Daiki Miki, Wataru Okamoto, Tomokazu Kawaoka, Masataka Tsuge, Michio Imamura, Kazuaki Chayama

**Affiliations:** 1 Department of Gastroenterology and Metabolism, Graduate School of Biomedical & Health Sciences, Hiroshima University, Hiroshima, 734-8551 Japan; 2 Cancer Treatment Center, Hiroshima University Hospital, Hiroshima, Japan; 3 Natural Science Center for Basic Research and Development, Hiroshima University, Hiroshima, Japan; 4 Collaborative Research Laboratory of Medical Innovation, Graduate School of Biomedical and Health Sciences, Hiroshima University, Hiroshima, Japan; 5 Research Center for Hepatology and Gastroenterology, Hiroshima University, Hiroshima, Japan; 6 RIKEN Center for Integrative Medical Sciences, Yokohama, Japan

**Keywords:** Hepatocellular carcinoma, Circulating tumor DNA, Lenvatinib

## Abstract

**Background:**

There has been a recent surge in interest in predicting biological effects associated with genomic alterations in order to implement personalized cancer treatment strategies. However, no reports have yet evaluated the utility of profiling blood-based circulating tumor DNA (ctDNA) in hepatocellular carcinoma (HCC) patients treated with lenvatinib (LEN).

**Method:**

We retrospectively performed ctDNA next-generation sequencing (NGS) analysis in 24 patients with advanced HCC at baseline and 4 weeks after initiation of LEN. Association of the changes in variant allele frequencies (VAFs) during treatment and clinical outcome were evaluated.

**Results:**

In total, 131 single nucleotide variants, 17 indels, and 23 copy number variations were detected as somatic alterations in 28, 6, and 12 genes, respectively in 23 of 24 patients. The most frequently altered genes were *TP53* (54%), *CTNNB1* (42%), *TERT* (42%), *ATM* (25%), and *ARID1A* (13%). The reduction in the mean frequency of variants (VAF_mean_) following 4 weeks of LEN treatment was associated with longer progression-free survival. The specificity and sensitivity of the reduction of VAF_mean_ for predicting partial response were 0.67 and 1.0, respectively, which were higher than those of serum α-fetoprotein level (0.10 and 0.93, respectively). No association between the mutation status at baseline and the effectiveness of LEN was observed.

**Conclusion:**

Our study demonstrated that somatic alterations could be detected in the majority of advanced HCC patients by ctDNA profiling and that ctDNA-kinetics during LEN treatment was a useful marker of disease progression. These results suggest that ctDNA profiling is a promising method that provides valuable information in clinical practice.

**Supplementary Information:**

The online version contains supplementary material available at 10.1186/s13046-021-02016-3.

## Background

Hepatocellular carcinoma (HCC) is one of the most common malignant tumors and is a leading cause of cancer-related death worldwide [1]. Lenvatinib (LEN) is an oral multikinase inhibitor that targets VEGF receptors 1–3, FGF receptors 1–4, PDGF receptor α, RET, and KIT [2]. Its approval is based on an international, multicenter, randomized, open-label, sorafenib non-inferiority trial (REFLECT; NCT01761266) that demonstrated a statistically significant improvement in progression-free survival (PFS) with LEN over sorafenib in patients with previously untreated, metastatic, or unresectable HCC [3]. More recently, an international, open-label, phase 3 trial (IMbrave; NCT03434379) demonstrated that treatment with atezolizumab plus bevacizumab was associated with significantly better overall survival (OS) and PFS outcome than sorafenib in patients with advanced unresectable HCC not previously treated with systemic therapy [4]. Consequently, atezolizumab plus bevacizumab was approved for the first-line treatment. While the number of drug options is increasing, the availability of biomarkers to predict treatment response to systemic therapies is limited to α-fetoprotein (AFP) for second-line ramucirumab [5]. Recently, associations between mutations in the PI3K/MTOR pathway and resistance to sorafenib as well as between mutations in the WNT pathway and resistance to immune checkpoint inhibitors have been reported [6, 7].

Molecular profiling has been gaining interest as a means to identify genomic alterations in cancer. The genetic landscape of HCC has been studied extensively [8–11]. However, the application of repeated biopsy in advanced HCC stages is limited for the following reasons: (i) the high specificity of radiological diagnosis; (ii) potential biopsy-related complications, such as bleeding and infection; (iii) and the limitation of single tissue biopsy in assessing tumor heterogeneity [12–14]. As an alternative, the use of circulating tumor DNA (ctDNA), also known as “liquid biopsy,” is a rapidly growing area of interest as a non-invasive test for the diagnosis and surveillance of cancer. Several studies have shown that ctDNA contains comprehensive information about tumor genomes, including variants originating from multiple independent tumors [15–17].

We have demonstrated that the relative level of ctDNA within a patient with HCC [18] or colorectal cancer [19] reflects the underlying tumor composition and that ctDNA levels change with time under therapeutic pressure. Several studies have shown that major somatic alterations in HCC, such as *TP53*, *CTNNB1*, *TERT*, and *ARID1A*, could be detected in ctDNA of HCC patients and that ctDNA has high specificity for detecting mutations in matched HCC tissue [20–24].

In various carcinomas, e.g., lung, bladder, and breast cancer, it has been reported that ctDNA kinetics can serve as a marker of therapeutic efficacy and can predict prolonged survival, suggesting that a change in VAF is directly related to antitumor activity and may have clinical significance [25–27].

On the other hand, there has been no report evaluating the utility of profiling blood-based ctDNA at baseline and/or during treatment with LEN in advanced HCC patients. The objectives of this study are as follows: 1) to evaluate the possibility of successful identification of ctDNA profiling using next-generation sequencing (NGS) in unresectable HCC patients treated with LEN; 2) to determine the utility of ctDNA in longitudinal monitoring of HCC treatment with LEN; and 3) to investigate the therapeutic implications of alterations that increase in frequency during LEN treatment by developing a search tool to identify therapeutic agents that might be effective against specific somatic alterations.

## Methods

### Patients

ctDNA was evaluated at the following two time points in 24 HCC patients (referred to as Hiroshima Guardant subjects HG1 through HG24) who had started LEN treatment at Hiroshima University Hospital between April 2018 and October 2019: (i) just before initiation of LEN and (ii) 4 weeks after initiation of LEN. The inclusion criteria were as follows: Child-Pugh liver function class A, an Eastern Cooperative Oncology Group performance status score of 1 or less, TNM stage 3 or higher, and a relative dose intensity > 70% during the initial 4 weeks. Patients were excluded when LEN treatment had been interrupted in the timeframe between the cfDNA profiling assays. Patients who received other treatments, such as transcatheter arterial chemo-embolization (TACE), during LEN treatment were censored. The end of follow-up was June 2020, and the median follow-up period was 14.3 months. Adverse events were graded according to the National Cancer Institute Common Terminology Criteria for Adverse Events version 4.0.

The study protocol was approved by the Hiroshima University ethical committee (approval numbers E-726-2 and HI-98) in accordance with the Declaration of Helsinki [28]. All patients provided written informed consent.

### Treatment regimens

Patients with a body weight of 60 kg or more started at an elevated dose of 12 mg once per day, while the remaining patients started at the standard dose of 8 mg once per day. Treatment interruptions and dose reductions were permitted in the event of adverse drug reactions. Twenty cases of LEN treatment were interrupted because of adverse events (4/20) or progressive disease (PD) (16/20). After discontinuation of LEN, 13 of the 20 patients underwent systemic post-LEN treatments, 2 underwent TACE, and 6 received best supportive care.

### Clinical and laboratory assessments

**Fig. 1:**
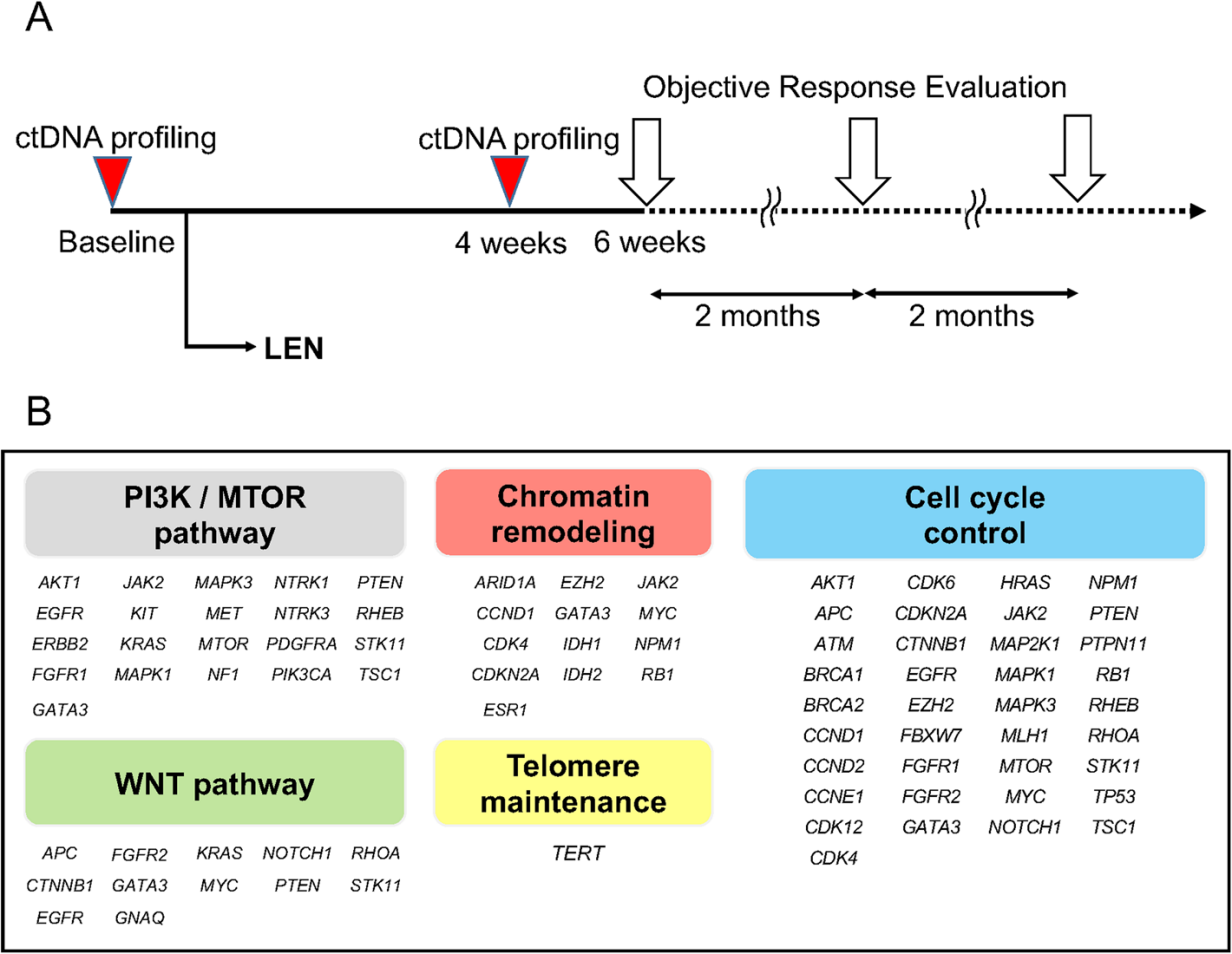
The timeline of the current study and comprehensive genomic classification. **A** The timeline of ctDNA profiling and objective response evaluation. ctDNA profiling was performed at baseline and after 4 weeks. Objective response was evaluated by mRECIST after 6 weeks of treatment and every two months subsequently. **B** Comprehensive genomic classification related to HCC included in the Guardant360 panel

Clinical and laboratory assessments were performed before treatment. Objective response was evaluated by modified Response Evaluation Criteria in Solid Tumors (mRECIST) [29] after 6 weeks (3–15 w) of treatment and every 2 months subsequently. Tumor size was evaluated based on a sum of the diameters (longest for non-nodal lesions, short axis for nodal lesions) for all target lesions, defined in RECIST 1.1 [30]. Plasma collected from each patient at baseline was aliquoted and stored at − 80 °C prior to ctDNA profiling as we previously reported [19]. In brief, 10 milliliters of peripheral venous whole blood were collected using EDTA as an anticoagulant at baseline and a median of 4 weeks (3–6 w) after the start of LEN treatment. Subsequently, the drawn blood was immediately processed to isolate plasma by a two-step centrifugation process: 3500 rpm for 10 min followed by 12,000 rpm for 10 min at 4 °C. Separated plasma was stored at − 80 °C. A timeline of the ctDNA profiling and objective response evaluation is shown in Fig. 1a. The primary endpoint of the study was PFS, and the secondary endpoint was OS.

### Next-generation sequencing

Cell-free DNA (cfDNA) was extracted from approximately 2 ml of plasma, and genomic alterations were analyzed as described by Lanman et al. [31] using Guardant360 v2.11, an NGS panel of 74 cancer-related genes utilizing Digital Sequencing of cell-free ctDNA. The Guardant360 method detects all four major variant classes (single nucleotide variants [SNVs] in all 74 genes; indels in 74 genes; copy number variations [CNVs] in 18 genes; and fusions in 6 genes). The targeted genes are shown in Supplementary Table 1. ctDNA sequencing, variant calling, and variant filtering were performed as described previously [32, 33] using a proprietary bioinformatics pipeline performed by Guardant Health. Manual review was performed at Guardant Health following variant calling. Minimal PCR amplification was conducted to ensure assay robustness and uniformity, and the limit of detectable VAF of the panel was 0.03% [32]. Single nucleotide polymorphisms (SNPs) and neutral variants registered in gnomAD and dbSNP databases were excluded using their proprietary bioinformatics pipeline [32].

### Genomic classification related to HCC

Patients were classified according to the mutations in major aberrant pathways of HCC. The following Gene Ontology and Reactome gene sets were used to determine the pathway component genes.

- WNT pathway: GO CANONICAL WNT SIGNALING PATHWAY and REACTOME BETA CATENIN INDEPENDENT WNT SIGNALING
- PI3K/MTOR pathway: GO PHOSPHATIDYLINOSITOL 3 KINASE SIGNALING and REACTOME MTOR SIGNALLING
- Cell cycle control: GO CELL CYCLE and REACTOME CELL CYCLE
- Chromatin remodeling: GO CHROMATIN REMODELING and REACTOME CHROMATIN MODIFYING ENZYMES (Fig. 1b).

### Definitions

VAF_mean_: The mean of the VAF(s) of somatic mutated genes in each patient. For example, given the following VAFs at baseline: gene A = 11%, gene B = 4%, gene C = 0%, and gene D = 0% at after 4 weeks: gene A = 12%, gene B = 4%, gene C = 2%, and gene D = 0%. In this case, the VAF_mean_ at baseline is (11 + 4 + 0) / 3 = 5, and after 4 weeks it is (12 + 4 + 2) / 3 = 6. Gene D is not detected at either time point, so it is excluded.

VAF_mean_-change: The change in VAF_mean_ during the treatment, calculated by subtracting VAF_mean_ at baseline from VAF_mean_ at 4 weeks.

### Statistical analysis

Statistical analysis was performed using JMP Pro 14.0.0 (SAS Institute Inc., Cary, NC, USA). Intergroup differences were tested using the Mann-Whitney U test or the Fisher’s exact test for continuous or categorical variables, respectively. Comparison of the VAF_mean_ between baseline and 4 weeks was assessed by paired t test. For continuous values, the median value was used as a threshold if no specific cutoff had been established. Sensitivity, specificity, positive predictive value (PPV), and negative predictive value (NPV) were calculated for the assessment of the diagnostic utility of the change in VAF_mean_ or tumor markers (serum AFP and des-gamma-carboxy pro-thrombin (DCP)). PFS and OS were estimated using Kaplan–Meier methods, and differences among subgroups were evaluated using the log-rank test. Univariate and multivariate Cox regression analysis was performed for potential biomarkers to predict PFS. All comparisons were considered significant if the *P* value was < 0.05.

### Validation

To address the lack of statistical power due to the small sample size and to reduce overfitting of the Cox regression model, we performed bootstrapping (B = 1000) using the rms 6.1–0 package in R version 4.0.2. We also used the R glmnet 4.1 package to calculate penalized hazard ratios using K-fold cross-validation to determine lambda.

### Matching variants with drugs of potential therapeutic effect

Translating ctDNA profiles to potentially actionable therapeutic strategies involves two steps. First, because most variants are expected to be benign or to have uncertain significance, the reported variants must be filtered to select variants of known or likely pathogenic effect. To this end, we compared predictions across several sources, including OncoKB [34], ClinVar [35], COSMIC [36], and CancerVar [37]. Second, because most variants have not been explicitly screened for their response to individual drugs, likely pathogenic variants were weighted based on evidence levels and specificity of the match. We searched for exact variant matches followed by broader partial matches based on the variant class against several databases, including CanDL (https://candl.osu.edu/), CIViC [38], Cancer Genome Interpreter [39], OncoKB [34], and CancerVar [37], filtering annotations based on the level of evidence, associated cancer type, approval status, and the predicted response to the therapy. An evidence report was then prepared based on review of the associated literature.

## Results

### Clinical characteristics

Patient demographics and baseline characteristics are shown in Table 1. The median PFS and OS were 6.4 months [95% confidence interval (CI), 4.8–8.4] and 17.8 months [95% CI, 13.2–22.4], respectively.

**Table 1:** Clinical characteristics of the patients

Variable	*n* = 24
Age	72.5 (54–88)
Sex (female/male)	6/18
Dose (8/12), mg	8/16
BCLC staging (B/C)	7/17
TNM staging (3/4a/4b)	7/4/13
T (0/1/2/3/4)	2/2/6/11/3
M (0/1)	12/12
N (0/1)	17/7
Main tumor size, mm	30 (0–135)
MVI (Vp2/Vp3/Vv)	1/1/0
AFP, ng/mL	10.3 (0.5–142,400)
DCP, mAU/mL	182.5 (13–37,535)
ALBI grade(G1/G2)	9/15
Platelet, ×10^4/μL	15.7 (5.7–144)
PT, %	85.5 (68–110)
Albumin, g/dL	3.6 (2.9–4.8)
Total bilirubin, mg/dL	0.7 (0.4–1.7)
AST, IU/L	32 (16–163)
ALT, IU/L	20.5 (6–91)
γGTP, IU/L	50.5 (11–183)
Etiology (HBV/HCV/NBNC)	5/11/8

*BCLC* Barcelona Clinic Liver Cancer, *TNM* Tumor, Node, Metastasis, *MVI* macroscopic portal vein invasion, *AFP* alpha-fetoprotein, *DCP* des-gamma-carboxy pro-thrombin, *ALBI* albumin-bilirubin, *PT* prothrombin time, *AST* aspartate aminotransferase, *ALT* alanine aminotransferase, *γGTP* γ-glutamyl transpeptidase, *HBV* hepatitis B virus, *HCV* hepatitis C virus, *NBNC* non-B non-C

### Genomic profiling of ctDNA

**Fig. 2:**
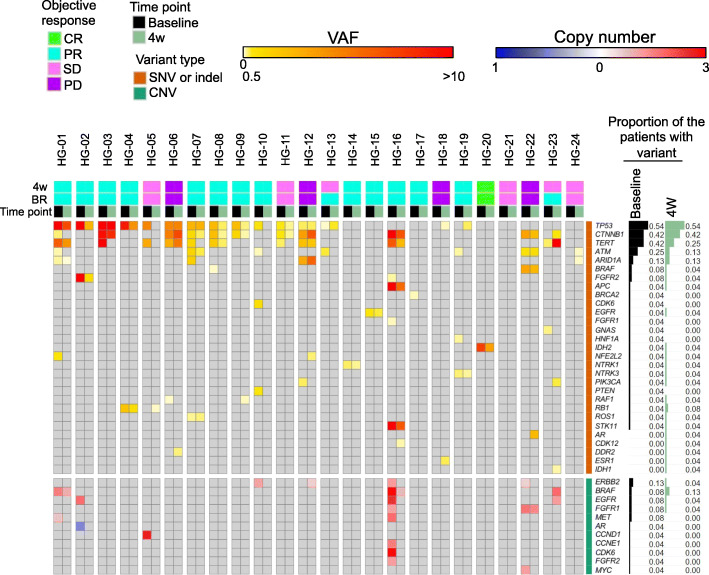
An overview of somatic alterations and changes detected in VAFs. **A** A heatmap showing the genomic profiling of ctDNA in 24 patients. SNVs or indels are shown in the upper block in a white (0%), yellow (0.5%), and red (> 10%) color scale. CNVs are shown in the lower block in a blue and red color scale. In cases in which multiple variants were detected in the same patient, the greater VAF/CNV is shown

**Fig. 3:**
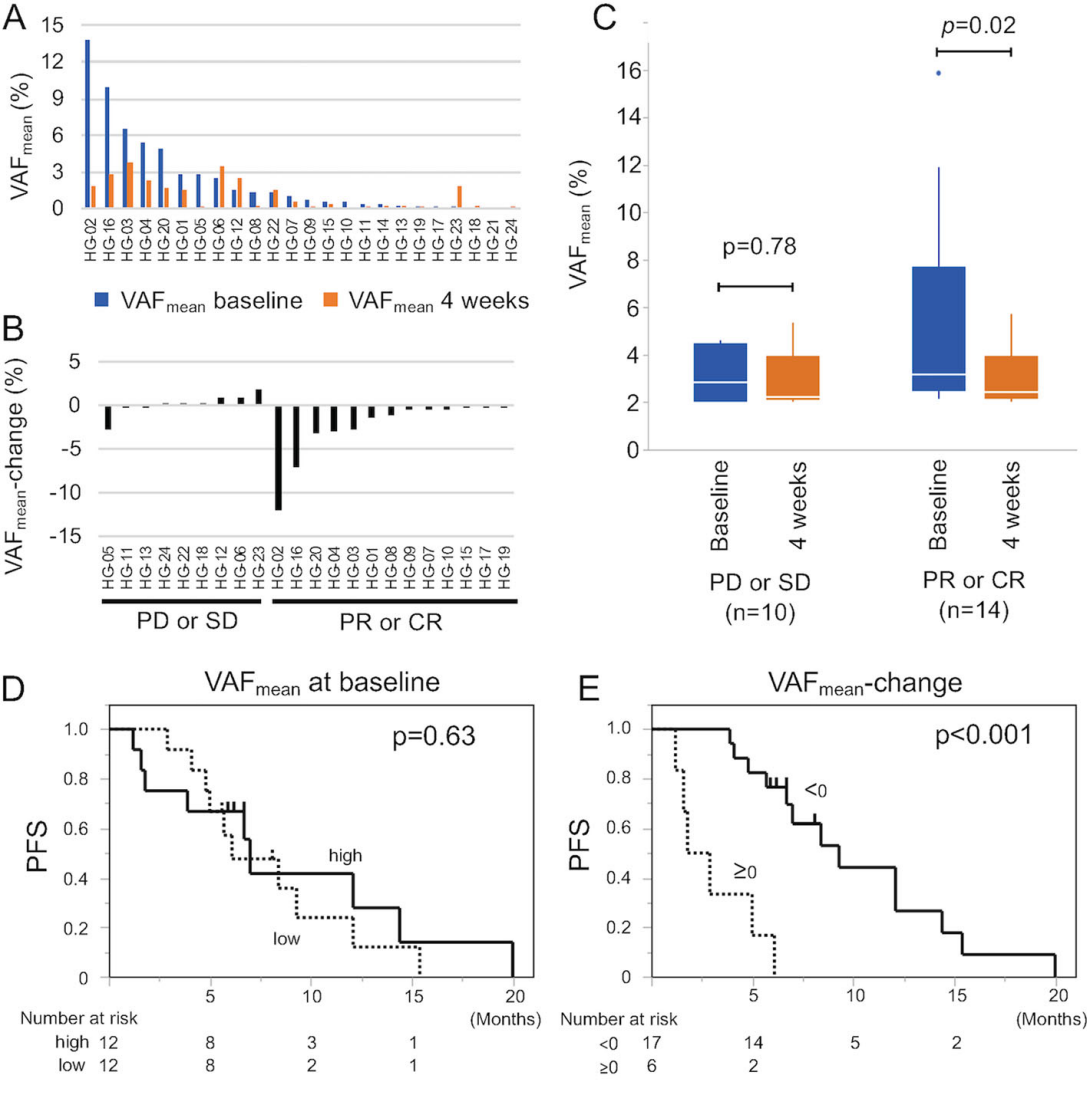
VAF_mean_ kinetics during the 4-week LEN treatment. **A** The distribution of VAF_mean_. Blue bars and red bars indicate VAF_mean_ at baseline and at 4 weeks, respectively. **B** The distribution of VAF_mean_-change. **C** Boxplot showing VAF_mean_ at baseline (blue) and at 4 weeks (red) according to the first objective response at 6 weeks. A paired t test was used for the comparison between the VAF at baseline and at 4 weeks. **D** Kaplan–Meier curve estimates of PFS according to VAF_mean_ at baseline. **E** Kaplan–Meier curve estimates of PFS according to VAF_mean_-change

In total, 131 somatic SNVs, 17 somatic indels and 23 CNVs were detected in 28, 6, and 12 genes in 23 of 24 patients, respectively (Fig. 2, Supplementary Table 2), suggesting that the method used has the sensitivity required for detecting ctDNA in most patients with advanced HCC. The percentages of patients with the top 5 somatic mutations at baseline were as follows: *TP53* (54%), *CTNNB1* (42%), *TERT* (42%), *ATM* (25%), *ARID1A* (13%). The distribution of VAF_mean_ is shown in Fig. 3a. VAF_mean_ increased between baseline and 4 weeks in 6/24 (25%) cases. The distribution of VAF_mean_-change is shown in Fig. 3b. Patient HG-21 was excluded from the analysis of VAF_mean_-change because no ctDNA was detected at baseline and after 4 weeks. The median value of VAF_mean_ was 0.85% (0–13.86). For the samples analyzed in this study, the depth of uniquely sequenced reads ranged from 952 to 10,459 (median, 4201).

Prior treatments are shown in Supplementary Table 3. Twenty-one patients had a history of prior treatment, and LEN was administrated as a first-line systemic treatment in 18 patients. There was no association between prior treatment and the number of mutations or the comprehensive genomic classification according to the mutated genes (Supplementary Table 4).

### Clinical characteristics of the patients according to the baseline VAF_mean_ and VAF_mean_-change

The patients were divided into a high VAF_mean_ group and a low VAF_mean_ group based on the median value of the VAF_mean_ at baseline. There was no significant difference in the clinical characteristics between the patients in the high and low VAF_mean_ group except for AFP (Table 2). There was no significant difference in clinical characteristics with respect to VAF_mean_-change.

**Table 2:** Clinical characteristics of the patients according to VAF_mean_ at baseline and VAF_mean_-change

	VAF_mean_ at baseline			VAF_mean_-change		
	high (≥0.85%^a^)	low (< 0.85%^a^)	p	< 0	≥0	p
sex (female/male)	2/10	4/8	0.6404	5/12	1/5	1
age, median	74 (67–84)	71.5 (54–88)	0.5826	71 (58–84)	76 (68–88)	0.1825
ALBI Grade, G1/G2, n	5/7	4/8	1	6/11	3/3	0.643
TNM, n						
4b/other	7/5	6/6	1	10/7	3/3	1
4/other	9/3	8/4	1	13/4	3/3	0.3185
T, n						
3 or 4/0–2	8/4	6/6	0.6802	10/7	4/2	1
4/0–3	1/11	2/10	1	2/15	1/5	1
M, 1/0, n	7/5	5/7	0.6843	9/8	3/3	1
N, 1/0, n	3/9	4/8	1	5/12	1/5	1
BCLC, B/C, n	3/9	4/8	1	4/13	3/3	0.3185
AFP,median, ng/mL	5.05 (0.5–1085.9)	193 (0.5–14,240)	0.0463	29 (0.5–142,400)	8.1 (0.5–1998.6)	0.441
DCP, median, mAU/mL	133.5 (13–37,535)	682 (14–16,575)	0.6033	143 (13–16,575)	1270.5 (27–37,535)	0.3627
MVI, presence/absence, n	1/11	1/11	1	2/15	0/6	1
History of prior treatment (yes/no), n						
Systemic therapy	3/9	3/9	1	4/13	2/4	0.6322
Catheter treatment	8/4	10/2	0.6404	13/4	4/2	0.6322
Local therapy	9/3	8/4	1	12/5	4/2	1
Radiation therapy	2/10	3/9	1	5/12	0/6	0.2725

*VAF* variant allele frequency, *ALBI* Albumin-bilirubin, *TNM* Tumor, Node, Metastasis, *BCLC* Barcelona Clinic Liver Cancer, *AFP* alpha-fetoprotein, *DCP* des-gamma-carboxy pro- thrombin, *MVI* macroscopic portal vein invasion

^a^The cut-off value of VAFmean at baseline set to median

### VAF_mean_ kinetics during the 4-week LEN treatment

Figure 3c shows VAF_mean_ at baseline and at 4 weeks according to the first objective response (6 weeks). In patients with PD or stable disease (SD), there was no significant difference in VAF_mean_ between baseline and 4 weeks (*p* = 0.78). On the other hand, in patients with partial response (PR) or complete response (CR), VAF_mean_ significantly decreased following 4 weeks of LEN treatment (*p* = 0.02). The specificity, sensitivity, PPV, and NPV of VAF_mean_-change < 0 for predicting best response PR and CR was 0.67, 1.0, 0.82, and 1.0, respectively (Table 3).

**Table 3:** The diagnostic utility of the change in VAF_mean_ or tumor makers

		mRECIST			diagnostic ability PR/CR			
		PD	SD	PR/CR	specificity	sensitivity	PPV	NPV
VAF_mean_	increase	4	2	0				
	decrease	0	3	14	0.67	1.0	0.82	1.0
AFP (all cases)	increase	1	0	1				
	decrease	3	6	13	0.10	0.93	0.59	0.50
AFP (≥20 ng/mL at baseline)	increase	0	0	0				
	decrease	2	1	8	0	1.0	0.73	–
DCP (all cases)	increase	4	5	10				
	decrease	0	1	4	0.90	0.29	0.80	0.47
DCP (≥40mAU/mL at baseline)	increase	4	4	6				
	decrease	0	1	3	0.89	0.33	0.75	0.47

*mRECIST* modified Response Evaluation Criteria in Solid Tumors, *AFP* α-fetoprotein, *DCP* des-gamma-carboxy pro-thrombin, *VAF* variant allele frequency, *PD* progressve disease, *SD* stable disease, *PR* partial response, *CR* complete response, *PPV* positive predictive value, *NPV* negative predictive value

### PFS and OS according to the VAF_mean_ at baseline and VAF_mean_-change

There was no significant difference in either PFS (Fig. 3d) or OS of patients with high or low VAF_mean_ at baseline, shown in Kaplan–Meier curves; *p* = 0.63 and *p* = 0.31, respectively. The median PFS (95%CI) and OS (95%CI) of patients with VAF_mean_ high/low were 7.0 (1.6–14.4) / 6.1 (4.1–12.1) months and 14.1 (7.2-not reached) / 21.5 (7.6-not reached) months, respectively. The median (95%CI) PFS and OS of patients with VAF_mean_-change < 0 / ≥0 following 4 weeks of LEN treatment were 9.3 (5.7–14.1) / 2.9 (1.2–183) months and 17.8 (13.2–21.2) months / not reached, respectively. Patients with VAF_mean_-change < 0 showed longer PFS (*p* < 0.001, Fig. 3e) than patients with VAF_mean_-change ≥0, but there was no significant difference in OS (*p* = 0.99).

### Correlation of tumor size, VAF_mean_ and tumor markers

**Fig. 4:**
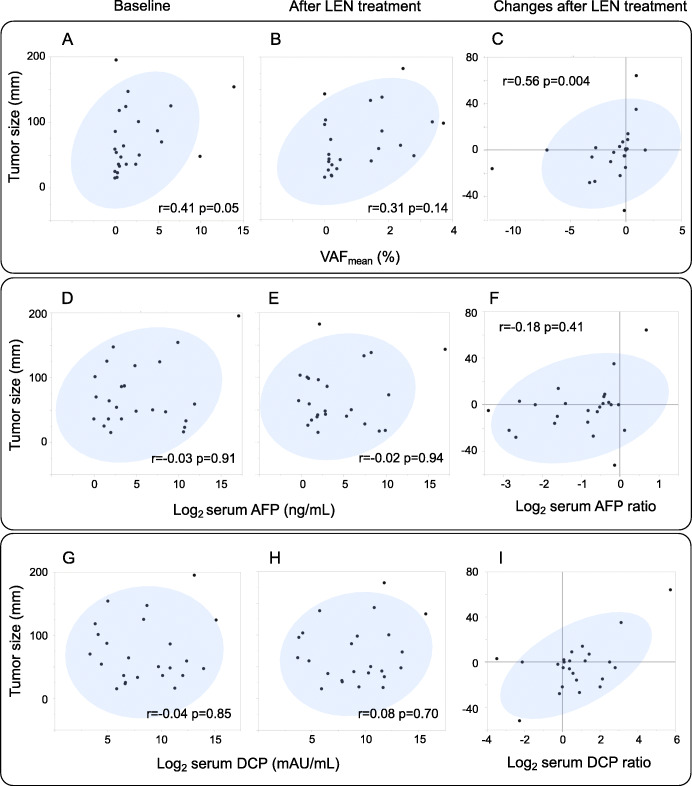
Correlation of tumor size, VAF_mean_ and tumor markers. A correlation between VAF_mean_ and tumor size at baseline (**A**) or after LEN treatment (**B**), and changes in tumor size and VAF_mean_-change(**C**) in Spearman’s rank correlation coefficient. A correlation between AFP/DCP and tumor size at baseline (**D**, **G**) or after LEN treatment (**E**, **H**). A correlation between changes in tumor size and in AFP/DCP before and after LEN treatment (**F**, **I**). Because the range of variation is large, the tumor markers are shown on a logarithmic scale (log_2_), and the change in tumor markers before and after LEN treatment is shown as a logarithmic scale (log_2_) of the ratio. After 4 weeks of LEN treatment, most cases showed a decrease in AFP (22/24, 92%) and an increase in DCP (19/24, 79%)

Tumor size was evaluated based on the sum of the diameters for all target lesions, defined in RECIST 1.1 [30], and shown in Supplementary Table 5. There was a positive Spearman’s rank correlation between changes in tumor size and VAF_mean_-change (*r* = 0.56, *p* = 0.004). The positive correlation was also observed between VAF_mean_ and tumor size at baseline (*r* = 0.41, *p* = 0.05) but not after LEN treatment (*r* = 0.31, *p* = 0.14) (Fig. 4a-c).

On the other hand, there was no significant correlation between tumor size and AFP or DCP at baseline or 4 weeks (Fig. 4d, e, g, h). Regarding the changes during LEN treatment, tumor size was positively correlated with DCP (Fig. 4i) but not with AFP (Fig. 4f).

### Comparison with tumor makers

AFP positive HCC was only 11/24 cases (45.8%). In all AFP positive cases (≥20 ng/mL at baseline), AFP was reduced by LEN treatment regardless of the results of the first response evaluation (Table 3). The specificity, sensitivity, PPV and NPV of the decrease of AFP for predicting PR and CR was 0.10, 0.93, 0.59, and 0.50, respectively, inferior to VAF_mean_-change < 0 (Table 3). There was no significant difference in either PFS (Supplementary Figure 1D) or OS of patients with decrease and increase AFP, shown in Kaplan–Meier curves; *p* = 0.30 and *p* = 0.16, respectively. The VAF_mean_-change may be better as early markers than changes in tumor markers.

Also in DCP, which is one of the important tumor markers in HCC, there was no significant difference in either PFS (Supplementary Figure 1E) or OS of patients with decrease and increase DCP, *p* = 0.80 and *p* = 0.24, respectively. The specificity, sensitivity, PPV and NPV of the decrease of DCP for predicting PR and CR was 0.9, 0.29, 0.8, 0.47, respectively (Table 3).

### Response to LEN according to specific mutations

**Fig. 5:**
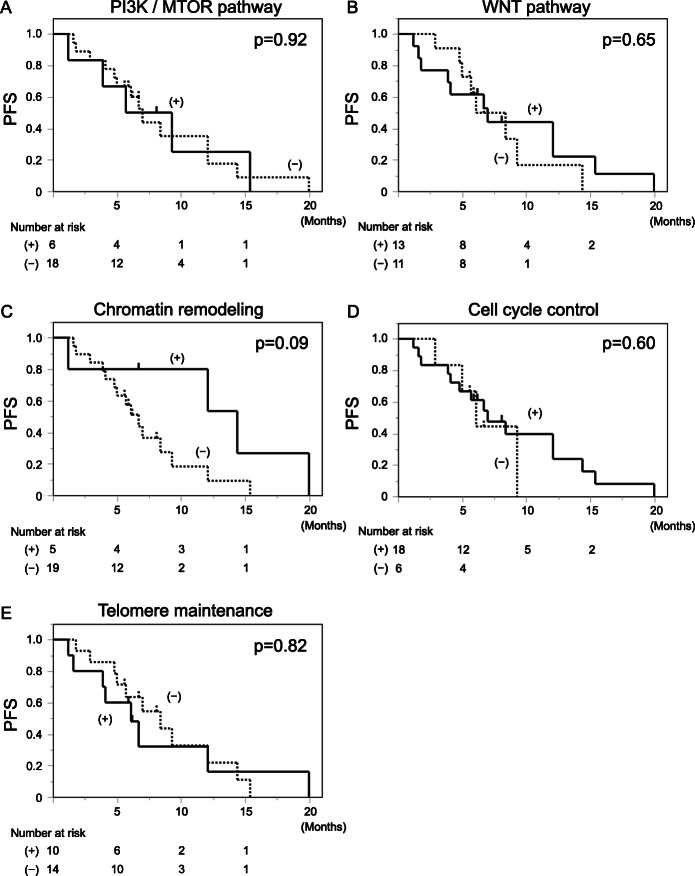
PFS according to the mutation in pathways frequently mutated in HCC patients. PFS according to the VAF_mean_ of PI3K/MTOR pathway (**A**), WNT pathway (**B**), chromatin remodeling (**C**), cell cycle control (**D**), and telomere maintenance (**E**) at baseline

Previous reports [6, 7] have shown that mutations in the PI3K/MTOR pathway were associated with resistance to tyrosine kinase inhibitors including sorafenib. We investigated the PFS according to the mutation in pathways frequently mutated in HCC patients, such as PI3K/MTOR pathway, WNT pathway, chromatin remodeling, cell cycle control and telomere maintenance. There were 6 patients with mutations related to PI3K/MTOR pathway, 13 patients with mutations related to WNT pathway, 5 patients with mutations related to chromatin remodeling, 18 patients with mutations related to the cell cycle, and 10 patients with mutations in the TERT promoter. Kaplan-Meier curves were drawn in PFS according to the status of the baseline somatic mutations of each classification (Fig. 5a-e), and no significant difference was found in all cases (*p* = 0.92, 0.65, 0.09, 0.60 and 0.82, respectively).

In the analysis of each of the top 3 genes at baseline, *TP53, CTNNB1,* and *TERT*, no significant differences in PFS were found (*p* = 0.09, 0.66 and 0.82, respectively) (Supplementary Figure 1A-C).

### VAF_mean_-change and response to treatment in different types of mutations

**Fig. 6:**
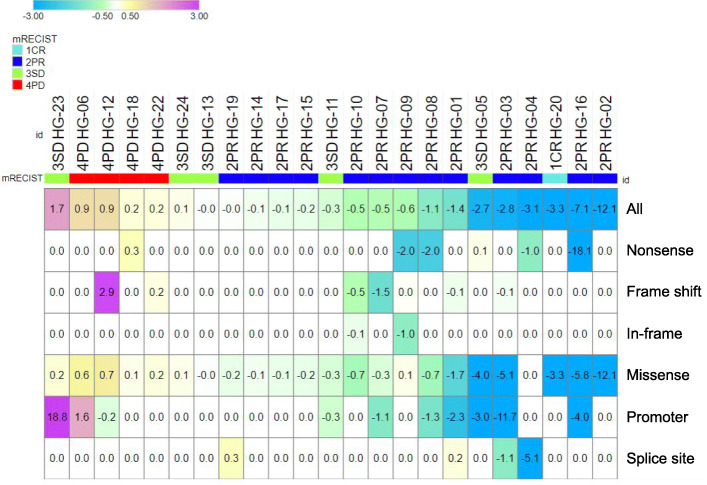
VAF_mean_-change in the different types of mutations. A heatmap showing the VAF_mean_-change in the different types of mutations including missense mutations, nonsense mutations, frame shift mutations, in-frame mutations, mutations in the promoter region, and splice site mutations. The cases are sorted according to the magnitude of the VAF_mean_-change in all mutations. Each VAF_mean_-change is shown in a blue/green color scale (< 0) and a yellow/purple color scale (≥0)

We classified the mutations into missense mutations, nonsense mutations, frame shift mutations, in-frame mutations, mutations in promoter regions, and splice site mutations. Figure 6 shows the VAF_mean_-change with respect to mutation type. The direction of the VAF_mean_-change was the same regardless of the mutation type in all but 5 patients. However, there were some mutations that changed in opposite directions. The VAF_mean_-change of missense mutations in HG-09, nonsense mutations in HG-05, splice site mutations in HG-01 and 19, and promoter region mutations in HG-12 showed the opposite direction from those of the remaining mutations. Each VAF_mean_-change in the opposite direction represented a VAF-change in a single gene mutation; *TP53* c.97-1G > A in HG-01, *RB1* R552* in HG-05, *TP53* L130F in HG-09, *TERT* c.-124C > T in HG-12, and *TP53* c.993 + 1G > T in HG-19.

### Factors associated with the cumulative incidence of poor PFS

The following parameters listed in Table 1 were analyzed by univariate analysis. AFP level was analyzed in two ways, the value at baseline and the change after 4 weeks of LEN treatment. Factors associated with poor PFS in univariate analysis were then analyzed by multivariable analysis.

Univariate analysis identified the following pretreatment factors associated with poor PFS: DCP (high/low, *p* = 0.03) and VAF_mean_-change (≥0/< 0, *p* < 0.001). Multiple Cox proportional hazard analysis identified only VAF_mean_-change ≥0/< 0 (HR 8.4, 95% CI = 2.3–31.2, *p* = 0.002) as an independent factor associated with poor PFS (Table 4). Results of bootstrapping using the rms package indicated a corrected Somer’s D statistic of 0.54 with optimism of 0.02 and a Harrell C index of 0.77. To calculate penalized hazard ratios, we performed 1000 iterations of 10-fold cross-validation with cv.glmnet to determine lambda (0.15). The penalized hazard ratio decreased from 8.4 to 4.8 for VAF_mean_-change ≥0/< 0 and from 2.3 to 1.6 for DCP (high/low).

**Table 4:** Prognostic factors for progression-free survival

Variable	Univariate	Multivariate		
	*p* value	*p* value	Hazard ratio	95% CI
VAF_mean_-change, ≥0/< 0	< 0.001	0.002	8.4	2.3–31.2
Age, high/low	0.52			
Sex, male/female	0.26			
Dose, 12 mg/8 mg	0.92			
BCLC, C/B	0.20			
TNM staging, 4b/3 or 4a	0.28			
T, 4/3 or less	0.05			
M, 1/0	0.50			
N, 1/0	0.35			
Main tumor size, high/low	0.95			
AFP, high/low	0.96			
AFP, decrease/increase	0.56			
DCP, high/low	0.03	0.13	2.3	0.8–6.7
ALBI, G2/G1	0.41			

*CI* confidence interval, *VAF* variant allele frequency, *BCLC* Barcelona Clinic Liver Cancer, *TNM* Tumor, Node, Metastasis, *MVI* macroscopic portal vein invasion, *AFP* alpha-fetoprotein, *DCP* des-gamma-carboxy pro-thrombin, *ALBI* albumin-bilirubin

### Assessment of alterations that increased during LEN treatment

In 10 patients, 22 SNVs in 12 genes and 2 indels in 2 genes were detected after 4 weeks of LEN treatment that had not been detected at baseline. An increase in the VAF of 15 SNVs in 6 genes and 2 indels in 2 genes were observed in 9 and 2 patients, respectively. Predicted associations between these variants and drugs with potential therapeutic efficacy are shown in Supplementary Table 6 (complete list) and Table 5 (extracted list of patients with PD at 6 weeks). In patient HG-06, a novel SNV appeared in *DDR2*, suggesting that the patient might respond to dasatinib. In patient HG-22, the emergence of *ATM* G2891D and increases in the VAFs of R2832H and S1905fs were observed, suggesting that drugs such as cisplatin, olaparib, and temozolomide might provide a potential therapeutic response. Patient HG-18 experienced emergence of *ESR1* R477*, suggesting that the patient might respond to fulvestrant but show resistance to exemestane. In patient HG-12, the frequency of *ARID1A* A2027fs increased, suggesting a potential response to ATR inhibitors, EZH2 inhibitors, and PARP inhibitors.

**Table 5:** A list of the alterations novelly appeared or increased during the lenvatinib treatment and the drugs with estimating effects for the gene alterations in patients with PD at 4 weeks

Gene	Variant_type	Indel_type	Mut_aa	Mut_nt	Patient_ID	Novelly detected or increased	Report	Evidence level	Drug	Estimating effects	Reference
*ARID1A*	Indel	Insertion	A2027fs	C > CA	HG-12	Increased	*ARID1A* oncogenic mutation	(CancerVar)	ATR inhibitors	Responsive	PMID:27958275
									EZH2 inhibitor	Responsive	PMID:25686104
									PARP inhibitor	Responsive	PMID:26069190
*CTNNB1*	SNV		G34V	G > T	HG-06	Increased	*CTNNB1* oncogenic mutation	(CancerVar)	Tankyrase inhibitor	Resistant	PMID:23539443
	SNV		S33C	C > G	HG-12	Increased					
*DDR2*	SNV		W778L	G > T	HG-06	Novelly detected	*DDR2* mutant	(CancerVar)	Dasatinib	Sensitive	PMID:22328973
*ESR1*	SNV		R477*	C > T	HG-18	Novelly detected	*ESR1* oncogenic mutation	Late trials: CGI|(CancerVar)	Fluvestrant	Responsive	PMID:27269946
								Late trials: CGI|(CancerVar)	Exemestane	Resistant	PMID:27269946
								LEVEL_3A:OncoKB	AZD9496		PMID:27986707|PMID:27269946|PMID:31563959
								LEVEL_3A:OncoKB	Fulvestrant		PMID:27986707|PMID:27269946|PMID:31563959
*TP53*	SNV		G245S	C > T	HG-06	Increased	*TP53* G245S	(CancerVar)	AMGMDS3	Resistance	PMID:25730903
	SNV		C238S	A > T	HG-18	Novelly detected	*TP53* oncogenic mutation	Early trials: CGI|(CancerVar)	Abemaciclib	Resistant	PMID:27217383
								Early trials: CGI|(CancerVar)	Cisplatin	Resistant	PMID:27646943
								Early trials: CGI|(CancerVar)	AZD6738	Responsive	NCT01955668|https://ash.confex.com/ash/2014/webprogram/Paper71027.html
								Early trials: CGI|(CancerVar)	Decitabine	Responsive	PMID:27959731
								(CancerVar)	Doxorubicin	Responsive	PMID:27397505
									Gemcitabine	Responsive	PMID:27397505
									Mitomycin C	Responsive	PMID:27397505
									WEE1 inhibitor	Responsive	PMID:25125259|PMID:27998224
									MDM2 inhibitor	Resistant	PMID:23084521|ASCO 2015 (abstr 10,564)
									Pramlintide	Responsive	PMID:25409149
*AR*	SNV		R609K	G > A	HG-22	Novelly detected	*AR* mutation	(CancerVar)	Nilutamide|Cyproterone Acetate|Flutamide|Bicalutamide	Resistance	PMID:26000489
*ATM*	SNV		R2832H	G > A	HG-22	Increased	*ATM* R2832H	LEVEL_1:OncoKB	Olaparib		PMID:32343890
*ATM*	SNV		G2891D	G > A	HG-22	Novelly detected	*ATM* oncogenic mutation	Early trials: CGI|(CancerVar)	Cisplatin	Responsive	PMID:26238431
*ATM*								Early trials: CGI|(CancerVar)	Olaparib	Responsive	ENA 2014 (abstr 8LBA)|PMID:26510020
*ATM*								(CancerVar)	ATR inhibitor	Responsive	ENA 2015 (abstr A48)
*ATM*	Indel	Insertion	S1905fs	T > TA	HG-22	Increased			Temozolomide	Responsive	PMID:23960094
*ATM*									DNA-PKc inhibitor	Responsive	PMID:23761041
*ATM*									PARP inhibitor	Responsive	ENA 2014 (abstr 8LBA)
*ATM*								LEVEL_4:OncoKB	Olaparib		PMID:20739657|PMID:26510020
*NFE2L2*	SNV		D29G	T > C	HG-12	Novelly detected					
*TERT*	SNV			C > G	HG-06	Increased					
	SNV			G > A	HG-06	Increased					

## Discussion

We previously reported that detection of ctDNA before surgery could predict microscopic vascular invasion of the portal vein and recurrence, especially extrahepatic metastasis within 2 years in HCC patients who underwent liver resection [18]. Several other studies have also examined the role of ctDNA as a predictive or prognostic marker [40–44]. For molecular-targeted therapies, some reports have shown that ctDNA is useful for evaluation of treatment response and resistance, such as *RAS* mutation in colorectal cancer or *EGFR* mutations in lung cancer, known as targeting driver genes [45–48]. In HCC, Oh et al. reported that the higher amount of cfDNA (total cfDNA) was associated with shorter time to progression and OS in HCC patients treated with sorafenib, but the VEGFA amplification was not significantly associated with treatment outcome [49]. However, to our knowledge, there are no reports related to changes in VAF during HCC treatment. The usefulness of the VAF_mean_ was previously reported by Raja et al., in which the reduction of VAF_mean_ was associated with longer PFS and OS in non–small cell lung cancer and urothelial cancer treated with durvalumab [27]. In our study, we demonstrated that the reduction of VAF_mean_ after initiation of LEN treatment for advanced HCC was associated with longer PFS. On the other hand, it was not associated with OS, possibly due to the effect of post-LEN treatments. In the OS analysis, 11 cases of censoring (survival cases within the follow-up period) were included, which may have affected the results. In the group showing VAF_mean_-change < 0, 7 out of 17 cases were censored, and in the group showing VAF_mean_-change ≥0, 4 out of 6 cases were censored. Although von Felden et al. recently reported that patients with mutations in the PI3K/MTOR pathway had significantly shorter PFS than those without these mutations after tyrosine kinase inhibitor treatment [7], no significant difference in PFS according to the presence of mutations in the PI3K/MTOR pathway at baseline was observed in our study. One reason might be that *TSC2*, which is involved in the PI3K/MTOR pathway and is frequently mutated in HCC, is not included in the Guardant360 panel used in our study. Another possible reason is that the therapeutic effects of LEN and sorafenib are different. In the study reported by von Felden et al., sorafenib was the most frequently used tyrosine kinase inhibitor (*n* = 18, 75%) [7].

A solid tumor consists of many sub clones with a range of different acquired mutations [12, 50]. It could be said that the response evaluated by the imaging to the treatment is synonymous with the response to the major clones in the tumor. Therefore, we considered that the VAF over a set of genes can reflect the response to the treatment with more precision. Zhang et al. suggested that on-treatment ctDNA kinetics are predictive of benefit with immune checkpoint blockade, which is a larger data set. They also had weighted the somatic SNVs and indels from the Guardant360 report equally when determining mean of VAF [51]. On the other hand, the analysis of the VAF_mean_-change within a particular mutation type revealed that there were minor populations that had a different direction of VAF change from the other major clones. That difference was considered to reflect the inequality of the responsiveness to LEN.

There was a positive correlation between the kinetics of the VAF_mean_ and the changes in the sum of the tumor diameters of the target lesions during LEN treatment. This finding supports the idea that VAF_mean_ level could serve as a non-invasive surrogate marker reflecting tumor burden. Serum AFP is the most widely used biomarker of HCC both for early diagnosis and evaluation of therapeutic efficacy and prognosis [52]. On the other hand, there was no correlation between tumor size and serum AFP nor between the change in the sum of the tumor diameters and the kinetics of AFP. There were also 6 patients who had discordant kinetics of AFP and VAF_mean_. For example, although patients HG-18 and HG-22 showed decreases in AFP (from 221 to 168 ng/mL and from 3834 to 1273 ng/mL, respectively) during the 4 weeks of LEN treatment, VAF_mean_ increased (from 1.27 to 1.44% and from 0 to 0.19%, respectively). They were diagnosed as PD at the first response evaluation, which matched the VAF_mean_ kinetics. In the remaining 4 patients, AFP had changed within a normal range (< 20 ng/mL). In addition, the specificity, sensitivity, PPV and NPV of the decrease of AFP or DCP for predicting PR and CR was inferior to those of VAF_mean_-change < 0. At least in evaluating the early response, the bottleneck in using AFP as a biomarker is that AFP had not been often positive and had tended to decrease after the start of LEN treatment in most cases (22/24, 92%). A systematic review showed that the sensitivity of AFP was 41–65% when using the commonly used positive cutoff value (AFP level ≥ 20 ng/mL) for HCC [53]. Another study investigated early tumor marker response and treatment response in patients with advanced HCC treated with LEN and concluded that the AFP levels of most patients had declined after 2 weeks, and by 4 weeks the group that had achieved a sustained reduction in AFP demonstrated a higher objective response [54]. This suggests that it is difficult to evaluate treatment response using AFP as a biomarker based on only one point. From these findings, it is possible that VAF_mean_-change might provide additional information to conventional tumor markers.

Although the variable timing of blood collection and imaging evaluation (Supplementary Table 5) is a major limitation of this study, the differences in the correlation between tumor size and VAF_mean_ at baseline and after 4 weeks could reflect the fact that antitumor effects that cannot be evaluated by tumor size alone, which supports the clinical usefulness of mRECIST.

This study suffers from several other important limitations: 1) a relatively modest cohort size, 2) no comparison with DNA from tumor, non-tumor liver and peripheral blood mononuclear cells, 3) non-uniform prior treatment history and variable timing of blood collection and imaging evaluation, and 4) a limited set of cancer-associated genes contained in the panel. Despite these limitations, our cohort represents a substantial effort to interrogate an uncommon but important clinical phenotype of ctDNA kinetics before and after LEN treatment. To compensate, in part, for the limited sample size and lack of a separate validation cohort for our multivariable Cox regression model, we performed internal validation with bootstrapping to estimate model over-optimism using the rms package and calculated penalized hazard ratios using LASSO regularization with the glmnet package. This approach does not obviate the need for independent validation of the results helps to reduce overfitting and suggests that the model may perform comparably with new data. Although the Guardant360 assay is not designed to distinguish between germline and somatic variants, ctDNA genotyping can distinguish germline mutations (present at ~ 50% VAF) from somatic mutations (present but typically at much lower VAF) [55, 56].

We used the definition of somatic mutations derived by Guardant’s analysis platform, in which high frequency mutations close to 50% are defined as germline mutation. Furthermore, the allelic frequency of a germline mutation detected in plasma will not change during treatment. Although that platform is currently widely used, we would like to mention that sequencing DNA from peripheral blood mononuclear cells could support to consider a mutation as somatic. Moreover, the mutations described in this study are in line with the genomic landscape of HCC [11, 57, 58]. The mutations in *TP53* were detected more frequently than in previous reports, but it has been demonstrated that advanced-stage HCC is associated with higher frequencies of *TP53* mutations [58] (Supplementary Table 7). Several reports have shown that somatic mutations can be observed in background liver [59–61]. However, clonal expansions in the case of cirrhosis have been reported to be millimeters in diameter [59], which suggests that the amount of ctDNA harboring the same mutation from background liver is less than that derived from the tumor region. For these reasons, we conclude that the majority of mutations detected in this study were derived from the tumor region. However, the possibility of a germline or background-liver origin cannot be completely excluded.

In our current cohort, prior treatment history was not associated with baseline genomic profiling or number of mutations. However, it is well known that mutation profile could change under therapeutic pressure [19, 62], so the effect should be considered non-negligible. Patient HG-23 (SD case with VAF_mean_-change ≥0) had a longer PFS of 6.1 months compared to PD cases (median PFS 1.7 months). Although patient HG-23 had been previously treated with durvalumab that had been discontinued due to interstitial pneumonia as an adverse effect, the antitumor effect persisted for a while without treatment. Prior immune checkpoint inhibitor therapy might have affected the PFS of LEN treatment. In patient HG-21, no variant was detected at either time point. A possible reason is that patient HG-21 had a smaller tumor size (Supplementary Table 3), and the amount of ctDNA may have been low. However, we cannot rule out that a mutation that was not included in the panel may have played a role. The Guardant360 panel does not contain several important HCC-associated genes such as *AXIN1/2* and *TSC2* [11, 57, 58]. More comprehensive platforms such as the 500 gene Guardant OMNI platform, which launched for research use only in 2017, or HCC-dedicated platforms could make it possible to analyze genes that are not yet included in the target gene list [63]. Therefore, it is necessary to balance cost-effectiveness with the selection of a panel that is well-suited to its intended purpose.

At least one somatic alteration in a cancer-related gene was detected in 23 out of 24 patients by ctDNA profiling using Guardant360 v2.11. Moreover, we were able to successfully match the newly emerged or elevated cancer-related variants with drugs that have already been approved for some types of cancer in 3 of the 4 patients with PD at 6 weeks. Although we have not established whether those mutations are associated with resistance to LEN treatment, these results suggest that ctDNA profiling may be useful to search for effective alternative therapies after progressive disease. Ikeda et al. reported that a patient with a *CDKN2A*-inactivating mutation and a *CTNNB1*-activating mutation received palbociclib and celecoxib treatment, and low levels of AFP were found at 2 months. Another patient with a *PTEN*-inactivating mutation and a *MET*-activating mutation received sirolimus and cabozantinib, and AFP was found to have declined by 63% (8320 to 3045 ng/mL) [23].

Four patients showed intrinsic resistance to LEN on PD after 6 weeks. In these patients, 7 SNVs in 5 genes that had not been detected baseline were increased after 4 weeks of LEN treatment: *TP53* R282W and C238S, *ESR1* R477*, *DDR2* W778L, *ATM* G2891D, *AR* R609K, and *NFE2L2* D29G. None of these genes is known to be associated with resistance to LEN treatment. ESR1 confers resistance to aromatase inhibitors [64, 65]. Discoidin domain receptors (DDRs), including DDR1 and DDR2, are two members of the collagen receptor family in the tyrosine kinase receptor subgroup. DDR1 activation by p53 induces the MAP kinase pathway and increases resistance to apoptosis [66], and other studies have demonstrated a chemo-resistant role of DDR1 activation in several cancers [67–69]. On the other hand, little is known about the role of DDR2 in the acquisition of tumor cell resistance to chemotherapy. Moreover, there is no hot spot for mutations in DDRs, and there is a lack of functional analysis of mutations in these genes [70]. ATM encodes a PI3K-related serine/threonine protein kinase (PIKK) and plays a central role in the repair of DNA double-strand breaks. Once activated, ATM phosphorylates many downstream effectors and causes cell-cycle checkpoint arrest, DNA repair, and apoptosis; hence it is thought that ATM plays a role in suppression of carcinogenesis [71]. Somatic mutations in *ATM* occur in many tumor types, particularly hematologic malignancies, and generally have been associated with inferior prognosis [71–73]. On the other hand, it has been reported that blockade of ATM improves the antitumor effects of sorafenib in HCC cells, with suppression of Akt signaling and significant potentiation of the cytotoxic effects [74, 75]. Involvement of this gene in LEN treatment resistance is unknown, but it may play a role.

On the other hand, in PD cases, *RAF1* K171R disappeared after 4 weeks of LEN treatment in patient HG-6, and *PIK3CA* G1007G disappeared in patient HG-12, who was naive to prior therapy. RAF1 is a kinase best known as the effector linking RAS to MEK/ERK activation. RAF1 has been reported as a negative regulator of hepatocarcinogenesis [76], although it has also been reported that RAF1 acts as an oncogene in HCC and that miR-4510 blocks HCC development through RAF1 targeting and RAS/RAF/MEK/ERK signaling inactivation [77]. Tian et al. reported that highly expressed RAF1 is associated with sorafenib resistance [78]. Interestingly, in the PR case HG-9, *RAF1* mutation had increased, and *CTNNB1* mutation had decreased following 4 weeks of LEN treatment, while in the PD case HG-6, *RAF1* had disappeared and *CTNNB1* had increased. Previous papers have revealed different modes of crosstalk between the two signals, WNT/β-catenin and RAS/MAPK, depending on the cellular context [79, 80].

Because most of the patients who require personalized systemic treatment are at advanced stages and their background is similar to that of the cohort of the present study, it could be said that this study was a simulation of clinical cancer genomics-based personalized treatment. Several recent studies have shown that β-catenin pathway activation represented by *CTNNB1* mutation was associated with an immune-cold microenvironment and resistance to immune checkpoint inhibitors in HCC patients [5, 81, 82]. On the other hand, our study suggested that the *CTNNB1* mutation status at baseline did not influence the effectiveness of LEN treatment. Further study is necessary to make conclusions. Considering our findings in light of previous studies, the assessment of mutations in genes in the β-catenin pathway, including *CTNNB1*, by ctDNA could be a useful indicator for treatment decisions; e.g., patients harboring such mutations might expect more benefit with LEN than immune checkpoint inhibitors.

## Conclusion

Our findings suggest that ctDNA profiling is well-suited for clinical cancer genomics with the following advantages: i) it is non-invasive, ii) it facilitates monitoring of changes in VAF over time, and iii) ctDNA kinetics may be provide additional information over conventional tumor markers.

## Supplementary Information

**Additional file 1.**

**Additional file 2.**

**Additional file 3.**

**Additional file 4.**

**Additional file 5.**

**Additional file 6.**

**Additional file 7.**

**Additional file 8.**

## Data Availability

All data generated or analysed during this study are included in this published article and its supplementary information files.

## Abbreviations

ctDNA: Circulating tumor DNA
HCC: Hepatocellular carcinoma
LEN: Lenvatinib
NGS: Next-generation sequencing
VAF: Variant allele frequency
SNV: Single nucleotide variant
CNV: Copy number variation
PFS: Progression-free survival
OS: Overall survival
AFP: α-fetoprotein
TACE: Transcatheter arterial chemo-embolization
PPV: Positive predictive value
NPV: Negative predictive value
RECIST: Response evaluation criteria in solid tumors
PD, SD, PR, CR: Progressive disease, Stable disease, Partial response, Complete response
cfDNA: Cell-free DNA
SNP: Single nucleotide polymorphism
95% CI: 95% confidence interval
