# Supplementary figures and images for: Identification and monitoring of mutations in circulating cell-free tumor DNA in hepatocellular carcinoma treated with lenvatinib
Source: J Exp Clin Cancer Res. 2021 Jun 26;40:215. doi: 10.1186/s13046-021-02016-3 (PMC8235843; doi:10.1186/s13046-021-02016-3)

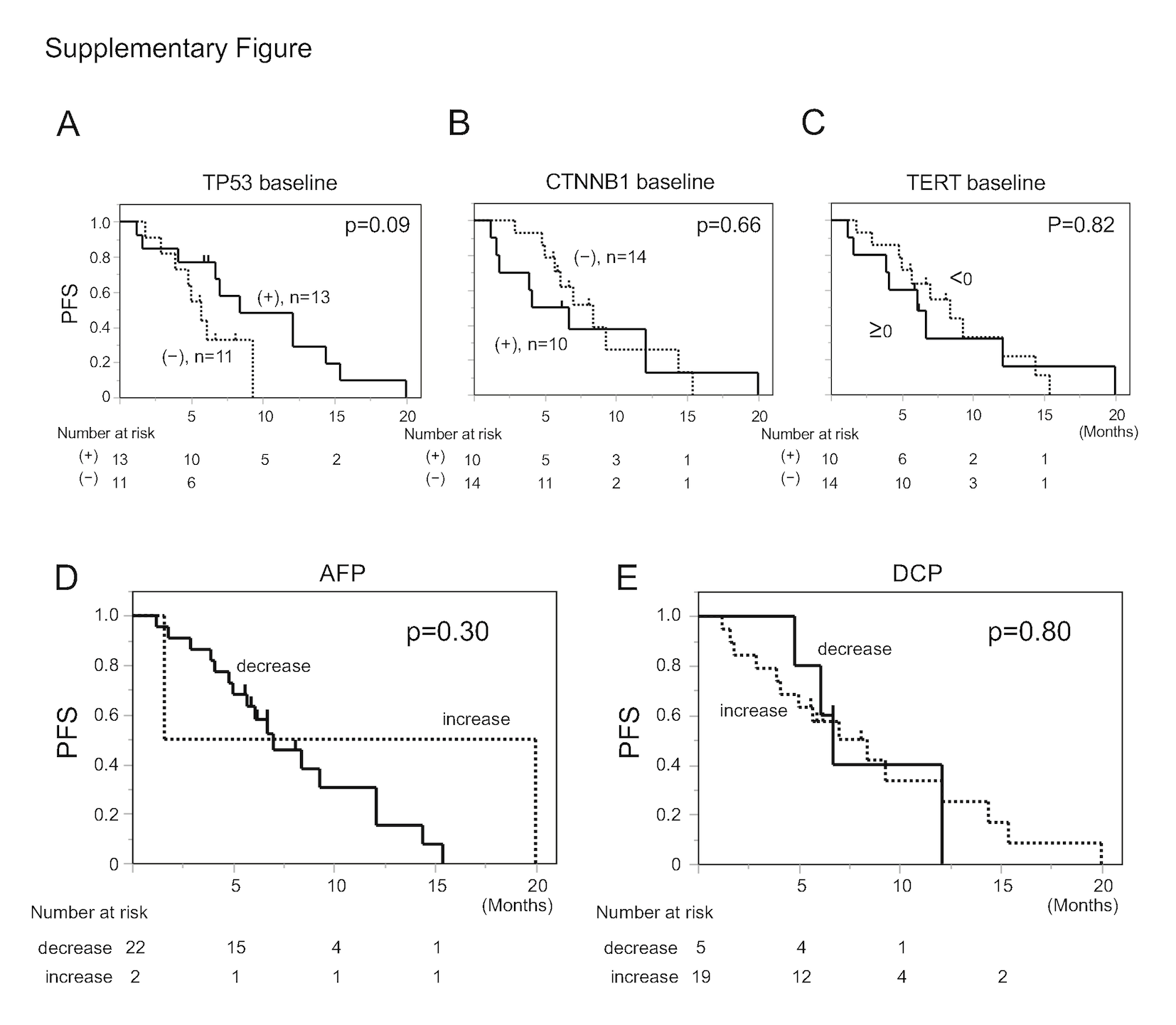

Supplement: Supplementary file 8 — Additional file 8. [file 13046_2021_2016_MOESM8_ESM.tiff]
